# Wearable Sensor and Mobile App–Based mHealth Approach for Investigating Substance Use and Related Factors in Daily Life: Protocol for an Ecological Momentary Assessment Study

**DOI:** 10.2196/44275

**Published:** 2023-04-11

**Authors:** Ayumi Takano, Koki Ono, Kyosuke Nozawa, Makito Sato, Masaki Onuki, Jun Sese, Yosuke Yumoto, Sachio Matsushita, Toshihiko Matsumoto

**Affiliations:** 1 Department of Mental Health and Psychiatric Nursing, Tokyo Medical and Dental University Tokyo Japan; 2 Department of Clinical Information Engineering, The University of Tokyo Tokyo Japan; 3 Department of Mental Health and Psychiatric Nursing, Osaka University Osaka Japan; 4 Humanome Lab, Inc Tokyo Japan; 5 National Hospital Organization Kurihama Medical and Addiction Center Yokosuka Japan; 6 Department of Drug Dependence Research, National Center of Neurology and Psychiatry Tokyo Japan

**Keywords:** alcohol and drug use, alcoholism, digital health, drug use, ecological momentary assessment, ecological momentary intervention, electronic health record, Fitbit, machine learning, mHealth, mobile app, self-monitoring, wearables devices

## Abstract

**Background:**

Digital health technologies using mobile apps and wearable devices are a promising approach to the investigation of substance use in the real world and for the analysis of predictive factors or harms from substance use. Moreover, consecutive repeated data collection enables the development of predictive algorithms for substance use by machine learning methods.

**Objective:**

We developed a new self-monitoring mobile app to record daily substance use, triggers, and cravings. Additionally, a wearable activity tracker (Fitbit) was used to collect objective biological and behavioral data before, during, and after substance use. This study aims to describe a model using machine learning methods to determine substance use.

**Methods:**

This study is an ongoing observational study using a Fitbit and a self-monitoring app. Participants of this study were people with health risks due to alcohol or methamphetamine use. They were required to record their daily substance use and related factors on the self-monitoring app and to always wear a Fitbit for 8 weeks, which collected the following data: (1) heart rate per minute, (2) sleep duration per day, (3) sleep stages per day, (4) the number of steps per day, and (5) the amount of physical activity per day. Fitbit data will first be visualized for data analysis to confirm typical Fitbit data patterns for individual users. Next, machine learning and statistical analysis methods will be performed to create a detection model for substance use based on the combined Fitbit and self-monitoring data. The model will be tested based on 5-fold cross-validation, and further preprocessing and machine learning methods will be conducted based on the preliminary results. The usability and feasibility of this approach will also be evaluated.

**Results:**

Enrollment for the trial began in September 2020, and the data collection finished in April 2021. In total, 13 people with methamphetamine use disorder and 36 with alcohol problems participated in this study. The severity of methamphetamine or alcohol use disorder assessed by the Drug Abuse Screening Test-10 or the Alcohol Use Disorders Identification Test-10 was moderate to severe. The anticipated results of this study include understanding the physiological and behavioral data before, during, and after alcohol or methamphetamine use and identifying individual patterns of behavior.

**Conclusions:**

Real-time data on daily life among people with substance use problems were collected in this study. This new approach to data collection might be helpful because of its high confidentiality and convenience. The findings of this study will provide data to support the development of interventions to reduce alcohol and methamphetamine use and associated negative consequences.

**International Registered Report Identifier (IRRID):**

DERR1-10.2196/44275

## Introduction

### Background

Substance use disorder (SUD) is a major public health concern worldwide. The harmful use of alcohol is responsible for 5.1% of the global burden of disease (7.1% for males and 2.2% for females) [1]. The prevalence of heavy episodic drinking, defined as ≥60 g of pure alcohol on at least one occasion at least once per month, was 18.2% in 2016 among the total population [2]. Alcohol use disorders are more prevalent in high-income countries [2]. About 5.5% of the global population aged 15-64 years had used psychoactive drugs in the previous year, and about 35 million people are estimated to be affected by drug use disorders [3]. However, treatment access has been limited because of the increasing costs of the health care system, patient time demands, and concerns about stigma [4-7]. Additionally, alcohol and other substance use increased during the COVID-19 pandemic [8-10]. The need for accessible and effective health care services has increased.

### Ecological Momentary Assessment

Advances in technology have the potential to reduce barriers to treatment services in this field. Broadly known as digital health or mobile health (mHealth), research in these areas uses mobile and computer software apps and wearable biosensor devices to understand and treat health conditions better [11-15]. In the field of SUD, various mobile apps have been developed to provide information on substance use and preventive health care services to continuously track specific health data such as substance use or support chronic condition management. Additionally, mHealth programs can help bridge treatment gaps by allowing patients to communicate with their physician or health care team without meeting face-to-face. One of the typical approaches of mHealth in this area is the ecological momentary assessment (EMA) method. The EMA approach has been developed to explore real-time substance use and motives, cravings, or triggers of substance use in daily life [12,16-19]. EMA has more benefits in capturing data on situations of substance use in the real world than cross-sectional or laboratory setting assessment because substance use is an episodic behavior influenced by immediate environmental factors (eg, external triggers) and internal factors such as stress and craving, which can be difficult to practically and ethically duplicate in an experiment environment [15-17]. Moreover, self-reported substance use can be biased by retrospective recall [20]. In a typical EMA, signaled prompts are sent to participants on a portable device (eg, smartphone) several times per day. The timing of the prompts depends on each study. There are generally the following 3 patterns: random time, fixed time, and event contingent. When participants receive these prompts, they are required to record their current thoughts, behaviors, and feelings on their devices.

Although EMA has advantages in assessing substance use accurately and repeatedly, compliance and missing data are still challenges [18,21]. Participants are likely to be unwilling to complete assessments during work, while studying, or when spending time with friends [22]. In people who use substances, noncompliance may occur because of an unstable life, comorbid psychopathology, and social pathology [17,23]. Missing data, especially systematically missing data (not at random), can lead to low statistical power and biased findings [18,24]. Uncomplicated EMA methods to minimize a participant’s engagement with a collection tool are needed to increase compliance with the EMA protocol. Moreover, it is difficult to confirm the validity of the data collected by self-reported assessment [25], even when using EMA methods, because social desirability bias influences subjective judgment when people report situations regarding substance use, especially illicit drug use [26,27].

### Wearable Activity Trackers

One of the solutions to the challenges of EMA might be the use of a wearable activity tracker (eg, Fitbit and Apple Watch), which can automatically collect participants’ data. Although these products are not specifically made for the data collection on substance use, researchers can repeatedly gather various objective data, such as behavioral data (eg, steps and sleep) and biological data (eg, heart rate, blood pressure, and degree of stress). These devices have been used in observational studies to collect accurate real-time data [28-30], as well as in intervention studies to promote healthy behavior and improve health-related outcomes [31-33]. Also, numerous data collected repeatedly over minutes, hours, or days for a certain period enable the prediction of health outcomes using machine learning methods. For example, acute exacerbation of chronic obstructive pulmonary disease [34], depression [35], movement types [36], sleep stage [37], and COVID-19 symptom exacerbation [38] were detected by analyzing data collected using wearable devices.

If a wearable activity tracker and a self-monitoring tool that records variables related to substance use are combined in a study, researchers can collect behavioral and biological data with minimal reliance on participant input. Moreover, researchers can analyze the data collected by a wearable activity tracker in many ways because these data could be potential predictive factors of substance use or subsequent outcomes following substance use. Daily self-monitored behavioral data along with momentary biological data collected by wearable activity trackers have the potential to bridge important data gaps.

### Purpose of This Study

This paper aims to introduce a procedure for implementing wearable tracker technologies as a method of EMA. We describe the methods to analyze data on substance use and psychological data using a self-monitoring app and objective behavioral and biological data using a wearable activity tracker (Fitbit). We will develop a predictive model of substance use by analyzing combined data using machine learning methods.

## Methods

### Materials and Equipment

#### Wearable Activity Tracker (Fitbit)

Fitbit (a registered trademark of Fitbit LLC) provides devices that offer a range of wearable activity trackers that can automatically track and display a users’ daily activity, exercise, and sleep data in real time. Fitbit is designed for use on both Android and iOS smartphones. Its popularity has grown consistently over the years, selling approximately 16 million devices in over 100+ countries in 2019, with an active user community of over 29 million users [39]. The device used in this study was the Fitbit Inspire 2 which uses a combination of a 3-axis accelerometer and optical heart rate monitor in order to track daily steps, calories burned, distance traveled, active minutes, floors climbed, sleep duration and quality, heart rate, and GPS-based information [40].

#### Self-monitoring Apps

Two self-monitoring mobile apps were newly developed for use in this study, depending on whether the participant had mainly alcohol or drug-related problems. Self-monitoring is a widespread approach in mHealth apps in the field of substance use [41]. Moreover, self-monitoring is low-intensive, feasible, and effective for reducing substance use [42]. App content in this study were developed based on previous studies that implemented a web-based relapse prevention program in which treatment effectiveness was already validated [43]. The newly developed self-monitoring apps used validated content and user interfaces from the previous study.

### Study Design

**Figure 1:**
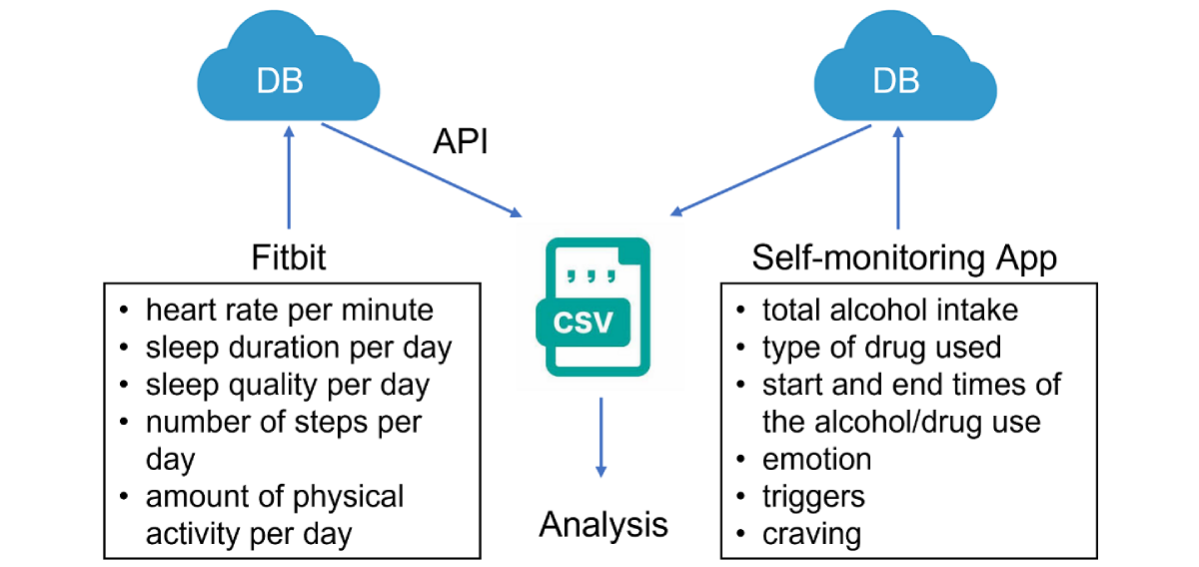
Content included in the self-monitoring apps. API: application programming interface; DB: database.

This study is an ongoing observational study using a Fitbit and a self-monitoring app (Figure 1). Participants were required to wear the Fitbit for 8 weeks as well as open the Fitbit app every day in order to sync the Fitbit data with their smartphones. Participants were also required to record daily entries in a self-monitoring app for 8 weeks and answer an in-app survey at the time of participation, 1-month, and 2-month follow-up. After the study was finished, participants received the Fitbit along with a gift card as compensation for participating in the study.

### Participants and Recruitment

Participants were outpatients with alcohol or methamphetamine use disorders from 2 psychiatric hospitals in Japan. A psychiatrist specializing in SUD screened potential participants based on inclusion and exclusion criteria, and patients were referred to the study if applicable. General residents with alcohol problems were recruited through a research company. Considering realistic circumstances at each facility and the approximate number of participants required to adequately perform preliminary model testing using machine learning, 20 people with methamphetamine use problems and 40 people with alcohol problems were recruited for this feasibility study. Inclusion criteria were as follows: (1) alcohol consumption with health risks in the past year (Alcohol Use Disorders Identification Test [AUDIT]: 8-19 points) [44,45], or methamphetamine use with health risks in the past year (Drug Abuse Screening Test [DAST]-10: 1-8 points) [46,47]; (2) owning a smartphone (iOS14 or Android 7.0 or later); (3) being able to wear the Fitbit constantly for approximately 8 weeks and record the self-monitoring app daily; and (4) being 20 years of age or older. Exclusion criteria were as follows: (1) not being able to speak or write in Japanese; (2) not being able to access the internet or complete the web-based survey; (3) not being able to participate for the entire 8-week research period, and (4) attending an outpatient clinic and having been deemed unsuitable to participate in the study by their attending physician.

### Measurements

#### Fitbit Data

The following data were measured while wearing the Fitbit for 8 weeks: (1) heart rate per minute, (2) sleep duration per day, (3) sleep stages per day, (4) the number of steps per day, and (5) the amount of physical activity per day. Data were synced from Fitbit to the Fitbit app every time it was connected to the participant’s smartphone. These data were retrieved from the Fitbit servers daily through an application programming interface and stored per user in a secure database created for this study. If there were no records for over 3 days in the past week, an email reminder was sent to the participant by the researcher.

#### Self-monitoring Data

**Figure 2:**
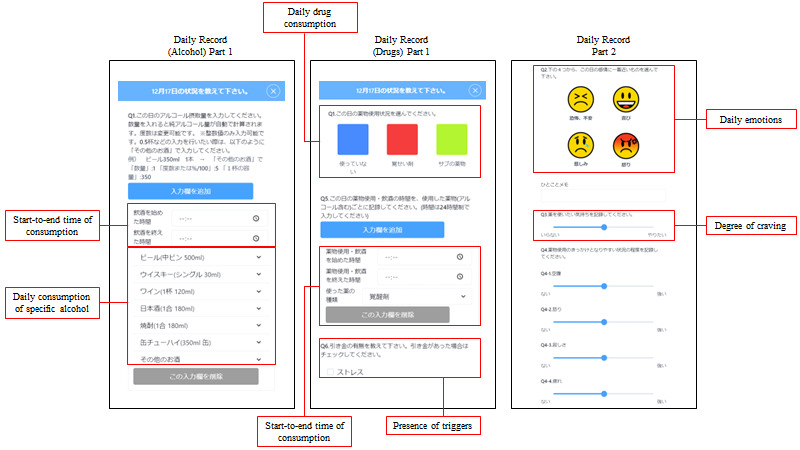
Framework for data collection using Fitbit and self-monitoring app.

The following data were measured daily through the self-monitoring app for 8 weeks (Figure 2): (1) total alcohol intake in pure ethanol equivalent format or the presence and type of drug used (depending on whether the participants’ main problem was alcohol or drug use), (2) general emotion of the day selected from 4 typical emotion symbols, (3) presence of alcohol or drug use triggers set by the participant, (4) degree of craving selected from a slider scale of 0-10 points, and (5) start and end times of the alcohol or drug use event. Data were retrieved from the self-monitoring app daily and stored per user in a secure database created for this study. Participants were advised to complete the daily entries at the end of each day, but were able to record entries the next day if it was difficult to record an entry before the end of the day.

#### Survey Data

##### Overview

Surveys were conducted at baseline, 4 weeks, and 8 weeks from the start of participation (Table 1). Alcohol- and drug-related measurements and demographics, as well as treatment history information were collected at the baseline survey. The 4-week survey consisted of the alcohol- and drug-related measurements as well as the usability and satisfaction measures, and the 8-week survey included alcohol- and drug-related measurements. A reminder was sent to the users’ registered email addresses when it was time to complete a survey, and a reminder email was sent after 3 days if the survey was not completed.

**Table 1:** Overview of measurements for each survey period.

		Baseline	4 weeks	8 weeks
**Alcohol- and drug-related measurements**				
	Amount of alcohol (grams of pure alcohol) or drug consumed per day in the past 7 days	✓	✓	✓
	Number of drinking or drug-use days during the observational period	✓	✓	✓
	University of Rhode Island Change Assessment Scale	✓	✓	✓
	General Health Questionnaire	✓	✓	✓
	Short Form-8	✓	✓	✓
**Usability and satisfaction**				
	System Usability Scale		✓	
	Client Satisfaction Questionnaire		✓	
	Adverse events concerning physical problems or discomfort using the app		✓	
**Demographics**				
	Gender, age, last education, employment status, and marital status	✓		
	Primary substance (alcohol or drug)	✓		
	Severity of substance use disorder (AUDIT^a^ or DAST^b^-10)	✓		
	Treatment history	✓		

^a^AUDIT: Alcohol Use Disorders Identification Test.

^b^DAST: Drug Abuse Screening Test.

##### Alcohol- and Drug-Related Measurements

The amount of alcohol or drug consumed per day (grams of pure alcohol or drug equivalent) in the past 7 days and the number of drinking or drug-use days during the observational period were assessed in this section. Mental health was measured by the General Health Questionnaire abbreviated version with a range of 0-30 points, validated in a Japanese population sample with a cutoff of over 6 points indicating mental distress [48,49]. Health-related quality of life measures were assessed by the Short Form-8, with higher scores indicating higher quality of life, validated in Japanese by previous studies [50,51]. Finally, the degree of behavioral changes was measured by the University of Rhode Island Change Assessment Scale (URICA) [52]. A higher URICA score indicates further progress in behavioral change. URICA was translated into Japanese, and equivalence between the English and Japanese versions was partially confirmed with back translation.

##### Usability and Satisfaction

The System Usability Scale (SUS), which consists of a total of 10 items, was used to assess the ease of use of the app [53,54]. A higher SUS score means good usability for the self-monitoring app. User satisfaction was measured using the Japanese version of the Client Satisfaction Questionnaire (CSQ) [55,56]. A higher score for the CSQ shows better satisfaction while using the self-monitoring app. Any adverse events concerning physical problems or discomfort using the app were asked in an open-ended question. The validity and reliability of SUS and CSQ have been confirmed in previous studies [54,56].

##### Demographics and Treatment History

Gender, age, last education, employment status, and marital status were asked as general demographic data. The following were also asked concerning the participants’ treatment history: the substance primarily responsible for the problem (alcohol, methamphetamine, marijuana, dangerous drugs, prescription drugs, over-the-counter drugs, organic solvents, or other drugs); the severity of SUD (AUDIT or DAST-10); the frequency of primary substance use in the past year; frequency of primary substance use in the past month (in days); the age of first alcohol or drug use; the age when first recognized an alcohol or drug use problem; the age when first sought medical care for an alcohol or drug use problem; social resources (medical institutions, mental health welfare centers, public health centers, or private addiction rehabilitation facilities); and the presence of comorbid psychiatric disorders and diagnosis.

### Data Analysis

**Figure 3:**
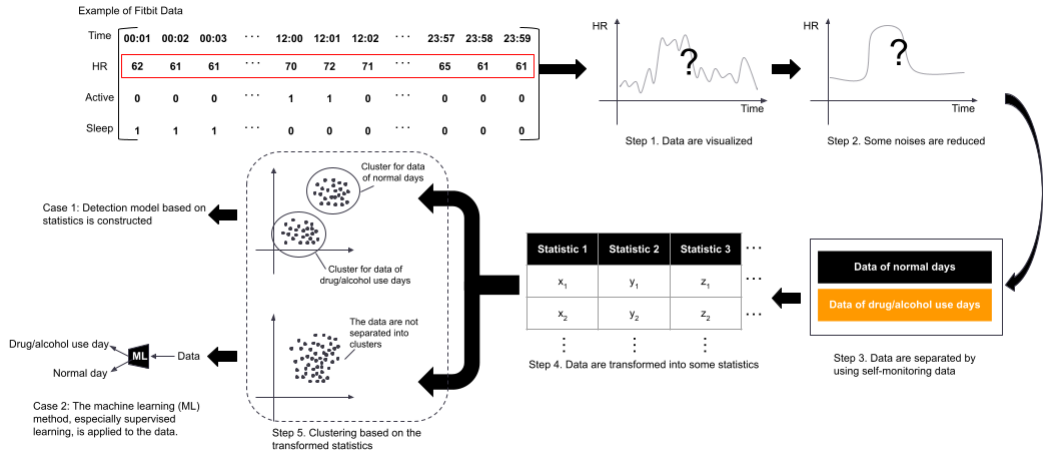
Planned future data analysis. HR: heart rate.

Fitbit’s application programming interfaces capture data for heart rate per minute, sleep duration per day, sleep stages per day, the number of steps per day, and the amount of activity per day (hereafter referred to as “Fitbit data”). We finished collecting the data in April 2021 and are preparing to conduct data analysis. In order to determine substance use based on the Fitbit data, 3 steps were applied, as shown in Figure 3, and are described in further detail as follows:

First, the Fitbit data will be visualized to confirm typical Fitbit data patterns for individual users. There are many patterns to detect drug or alcohol use; for example, the heart rate may remain high while drinking alcohol. Accordingly, data visualization is beneficial for confirming such patterns. If, for example, we can detect a typical pattern as mentioned above, signals other than that pattern may be considered noise for this analysis. Subsequently, noise removal methods will be applied to the Fitbit data. For example, average heart rate or heart rate variability may also be influenced by exercise or walking rather than drug or alcohol use. Therefore, we will use step counts or activity amounts on Fitbit data to specifically filter out such movement or workout periods. Other possible methods for noise removal, in terms of signal processing, may include signal smoothing methods, for example, moving average and Gaussian filter. Methods will be chosen to retain typical patterns but remove the other signals.

Second, the Fitbit data will be analyzed by using statistical analysis methods in order to predict drug or alcohol use on a relatively long-term basis, such as within a day or a week. By using self-monitoring data, we can separate the Fitbit data into data reflecting substance use and data from normal activity. Some descriptive statistical methods, for example, averaging, variance, self-correlation, or frequency-domain analysis, will be applied to each parameter in the separated data sets to determine features that include the days a user has declared drug or alcohol use. In this analysis, survey data containing the participants’ relation to drug or alcohol use, along with psychological and behavioral data points, may also be added as complements through the descriptive statistics. After transforming the data into its statistics, we would like to apply clustering methods to provide a quantitative evaluation of typical patterns reflecting drug or alcohol use.

There are 2 approaches to determining an optimal detection method for drug or alcohol use. If we can successfully obtain distinctive features by using the above statistical analysis, we will accordingly construct a detection model based on it. However, constructing such a model may be insufficient to isolate use with high accuracy and precision. Therefore, data analysis using both machine learning and statistical analysis methods will be performed in order to create a model that solves the problem as indicated above, based on the combined Fitbit and self-monitoring data. The primary candidate for machine learning methods will be Long Short-Term Memory. Long Short-Term Memory is considered one of the most suitable methods for analyzing time series data, which applies to this study. Meanwhile, other deep learning methods will also be considered to fit the data. The deep learning model will be carefully selected after we perform the statistical analyses, and then this model will be tested based on 5-fold cross-validation. Partitioning data depends on the degree of temporal precision with which drugs and alcohol consumption is estimated. Further preprocessing and machine learning methods will be conducted based on the preliminary results of the validation.

### Ethical Considerations

This study was approved by the Ethics Committee of the Faculty of Medicine and Graduate School of Medicine of Tokyo Medical and Dental University (M2020-189) and the Institutional Review Boards of each recruiting hospital. Data collected from the self-monitoring app were automatically stored in a protected database. Fitbit data were archived from the Fitbit server and preprocessed on a cloud-based, protected database without personal information attached. These data were managed by Humanome Lab, Inc. Both self-monitoring data and Fitbit data were then matched using participants’ ID numbers. Patients or participants provided their written informed consent in person before participating in this study.

## Results

Enrollment for the trial began in September 2020, and the data collection was completed in April 2021. In total, 13 people with methamphetamine use disorder and 36 with alcohol problems participated in this study. Table 2 shows participants’ demographics and days of methamphetamine use or drinking. The mean age of the participants was approximately 46 years. In the methamphetamine group, most participants were male, and 5 (38%) were unemployed. In the alcohol group, half of them were male, and 26 (72.2%) were employed. The severity of methamphetamine or alcohol use disorder assessed on the DAST-10 or AUDIT-10 was moderate to severe. The resting heart rate measured by Fitbit was 72 in the methamphetamine group and 65 in the alcohol group, whereas the sleep duration per day was 408 minutes in the methamphetamine group and 386 minutes in the alcohol group.

There are 2 main anticipated results in this study. The first is to gain a better understanding of physiological and behavioral data before, during, and after alcohol or methamphetamine use. For example, an extended period of sleep deprivation may lead to a heightened sense of craving, and alcohol or methamphetamine use may increase. On the other hand, sleep quality may decrease due to several days of drinking.

The second anticipated result is to develop more personalized predictive models for alcohol or methamphetamine use. Data gathered through Fitbit are valuable in that it provides longitudinal and sequential data compared to periodic visits to hospitals. Although Fitbit is not a medical device, these data offer a new perspective into the daily life of participants with alcohol or methamphetamine problems. For example, individual participants may drink at night on workdays when stressed, and their heart rate increases to 110 beats per minute during the day.

In the future, understanding physiological and behavioral data and identifying individual patterns of behavior may lead to more effective personalized interventions that support preventive behaviors such as promoting eating or sleeping as a method of harm reduction when there is the detection of continuous levels of high stress and accelerated heart rate. Limitations of previous EMA and wearable tracker studies are still prevalent in this study, as compliance and missing data are still predictable risks. However, along with traditional deterrence methods such as data visualization, rewards, and reminders, this study uses consecutive biological data collected through Fitbit and an extended study period of 8 weeks to supplement missing data.

**Table 2:** Demographic characteristics of participants.

			Drug use disorder (N=13)		Alcohol use disorder (N=36)
Sex (male %), mean (SD)			12 (92.3)		18 (50)
Age (years), mean (SD)			46.9 (9)		45.5 (10.7)
**Education, mean (SD%)**					
	Middle school	3 (23.1)		1 (2.8)	
	Secondary school	4 (30.8)		6 (16.7)	
	College	1 (7.7)		6 (16.7)	
	University	5 (38.5)		23 (63.9)	
**Employment, mean (SD%)**					
	Employed	5 (38.5)		26 (72.2)	
	Unemployed	5 (38.5)		3 (2.8)	
	Other	3 (23.1)		7 (19.4)	
**Marital status, mean (SD%)**					
	Married	11 (84.6)		10 (27.8)	
	Single	2 (15.4)		22 (27.8)	
	Divorced	0 (0)		4 (27.8)	
Age at first drug or alcohol use (years), mean (SD)			21.2 (6.4)		18.8 (2)
Age at first hospital encounter (years), mean (SD)			39.9 (9.3)		—^a^
**DAST^b^-10**					
	Score, mean (SD)	6.9 (1.4)		—	
	Moderate level (3-5; %), mean (SD)	3 (23.1)		—	
	Substantial level (6-8; %), mean (SD)	8 (61.5)		—	
	Severe level (9-10; %), mean (SD)	2 (15.4)		—	
**AUDIT^c^-10**					
	Score, mean (SD)	—		13.8 (5)	
	Low-risk consumption (0-7; %), mean (SD)	—		4 (11.1)	
	Hazardous consumption (8-14; %), mean (SD)	—		17 (47.2)	
	Alcohol dependence (15-40; %), mean (SD)	—		15 (41.7)	
**Days of drug use, mean (SD)**					
	Did not use	35.9 (17.3)		—	
	Used primary drug	10.4 (14.9)		—	
	Used secondary drug	0.3 (0.6)		—	
	Did not answer	2.2 (5.5)		—	
	No record	7.2 (12.3)		—	
Days of drinking, mean (SD)			—		33.1 (17.8)
Drinking amount per day (g), mean (SD)			—		75.6 (65.5)
**State of mind (days), mean (SD)**					
	Good	20.5 (11.3)		31 (13.9)	
	Not good	16.8 (13.2)		4.3 (6.3)	
	Angry	2.8 (3.5)		2 (3.1)	
	Sad	5.5 (5.5)		3.1 (4.3)	
	Did not answer	3.1 (5.6)		3.8 (7)	
	No record	7.2 (12.3)		11.9 (12.8)	
Craving (0-10)**,** mean (SD)			4 (3.1)		4.6 (1.9)
Resting heart rate (beats per minute)**,** mean (SD)			72.6 (8)		65.9 (9.4)
Sleep duration per day (minutes)**,** mean (SD)			408.3 (192.8)		386.4 (100)
**Sleep stages (minutes), mean (SD)**					
	Wake	57.7 (35.8)		55.8 (22)	
	Light sleep	242.9 (128.6)		223.1 (66.8)	
	Deep sleep	73.8 (40.8)		66.4 (25)	
	REM^d^ sleep	82.1 (49.4)		88.1 (36.5)	
Steps per day**,** mean (SD)			8884.9 (6450.2)		10,277 (5481.2)
**Physical activity per day (minutes), mean (SD)**					
	Lightly active	204.8 (113)		233.7 (100.5)	
	Fairly active	34.3 (55.6)		19.7 (23.4)	
	Very active	38.6 (78.8)		28.4 (31.2)	

^a^Not available.

^b^DAST: Drug Abuse Screening Test.

^c^AUDIT: Alcohol Use Disorders Identification Test.

^d^REM: rapid eye movement.

## Discussion

In this study, we collected data related to substance use and consecutive biological and behavioral information simultaneously using Fitbit and a smartphone app. To the best of our knowledge, this study is the first to collect and analyze real-time data on daily life among people with substance use problems in Japan. Similar to other Asian countries, drug policies in Japan are strict, and there is severe stigmatization against people with substance use problems. The new approach to data collection in this study might be helpful because of its high confidentiality and convenience. We will analyze the data in 2023 and then recruit additional participants based on the results for a more robust analysis.

The use of digital health technologies will be increasingly necessary in the future. In fact, during the COVID-19 pandemic, innovations in cost-effective and user-friendly drug prevention and treatment services, such as internet-based or mobile phone-based services, accelerated to increase accessibility and coverage of services [10]. Accordingly, in future research, based on the findings of this study, we will develop an ecological momentary intervention program to encourage participants to reduce substance use and negative consequences.

## Abbreviations

AUDIT: Alcohol Use Disorders Identification Test
CSQ: Client Satisfaction Questionnaire
DAST: Drug Abuse Screening Test
EMA: ecological momentary assessment
mHealth: mobile health
SUD: substance use disorder
SUS: System Usability Scale
URICA: University of Rhode Island Change Assessment Scale

## Data Availability

The data sets in this study are not available because we have not obtained the agreement of participants to disclose raw data or the approval of the ethics committee in each institution for data sharing.
